# Catheter ablation of Atrial Incisional Tachycardia mistaken for Atrial flutter

**Published:** 2007-04-01

**Authors:** L Ottaviano, C Muto, G Carreras, M Canciello, B Tuccillo

**Affiliations:** 1 Department of Cardiology, S. M. Loreto Mare Hospital, Naples, Italy; 2 Department of Cardiology, S. Luca Hospital, Vallo della Lucania (SA), Italy

**Keywords:** incisional tachycardia, radiofrequency ablation, electroanatomic and activation mapping system

## Abstract

Incisional sustained tachycardias are frequent in patients who have undergone a surgical repair of interatrial defect. A 43-year-old woman with drug refractory, highly symptomatic, persistent atrial tachycardia in the last year, was referred to our unit for catheter ablation. The patient had undergone a cardiac operation for repairing interatrial secundum ostium type defect with a patch five years before. A previous radiofrequency ablation procedure had been performed for common atrial flutter. We describe a case of incisional  atrial tachycardia ablation guided by the new EnSite NavX system equipped with a new electroanatomic mapping system.

## Background

Incisional sustained tachycardias are frequent in patients who have undergone surgical repair of  an interatrial defects [1,2]. The most common electrophysiological mechanism is macro re-entry around a scars [3]. In most cases these arrhythmias are incessant and drug refractory. Hence a radiofrequency ablation (RF) procedure is a right  therapeutic choices [4-9]. We describe a case of atrial incisional tachycardia ablation guided by the new EnSite NavX system (Endocardial Solutions Inc., St Paul, Minnesota) equipped with a new electroanatomic and activation mapping systems [10-12].

## Clinical history

A 43-year-old woman with drug refractory, highly symptomatic, persistent atrial tachycardia in the last year, was referred to our unit for catheter ablation. Five years ago the patient was undergone  a cardiac operation for repairing an interatrial secundum ostium type defect. ECG showed an atrial tachycardia whose features resembled a common atrial flutter. Echocardiography images showed no  significant atrial dilation, no residual interatrial shunt and a normal ventricular systolic function.

## Electrophysiological study (EP) and Ablative procedure

A quadripolar Josephson diagnostic catheter was inserted into the coronary sinus via left subclavian vein as a reference. A bipolar Cournard diagnostic catheter was inserted into the right ventricular apex. A  20-pole steerable catheter was placed around the tricuspid annulus. An 8 mm tip catheter (Boston Scientific EP Technologies) was used for mapping and ablation. The electrophysiological findings showed a stable atrial tachycardia of 230 ms cycle length which was firstly misunderstood as a common atrial flutter (counter clockwise) showing a caudocranial septal activation and craniocaudal activation along the right lateral atrial wall. The ablation catheter was therefore positioned on the cavo-tricuspid (CT) isthmus, considering this region crucial for the macroreentry circuit. But overdrive pacing at 210 ms cycle length from CT isthmus did not show any concealed entrainment. So we were inclined to believe that it was an incisional tachycardia and therefore we performed a reconstruction of the three-dimensional geometry of the right atrium  using the EnSite NavX system.

**Figure 1:**
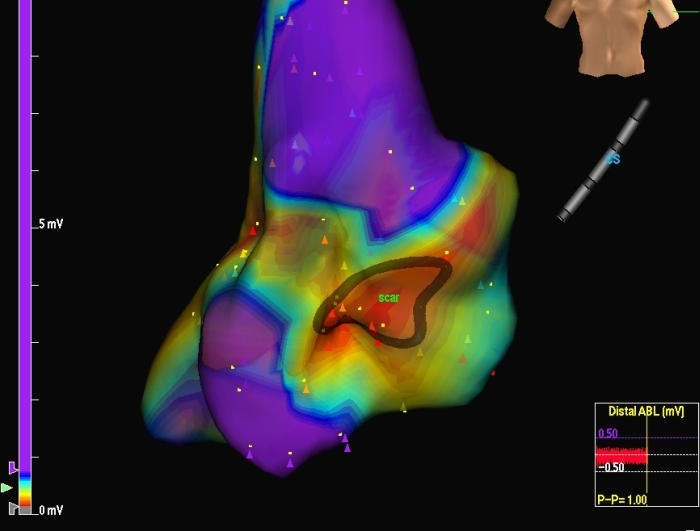
A large no - signal area was present on interatrial septum. The absence of local electrograms in this region corresponded to a large patch used for repairing interatrial defect (scar).

An activation-voltage map was obtained moving the ablation catheter point by point inside the right atrium while the patient was still in stable atrial tachycardia. The voltage map showed a large no-signal area  on interatrial septum corresponding to a large patch used for repairing an interatrial defect (Figure 1). A large macroreenty with a counter-clockwise pattern was displayed around this area of no-local electrograms. Lateral right atrial wall and CT isthmus region were probably activated as by-stander because no concealed entrainment was detectable from these areas. A narrow area of slow conduction velocity was clearly identified between the patch and the tricuspid  valve annulus, posterior and inferior to the coronary sinus ostium. The stimulation of this  narrow area showed concealed entrainment of the atrial tachycardia.

RF application  in continuous way (70 Watts 55 °C) was then attempted along this isthmus drawing back the ablation catheter from the scar area to the tricuspid valve annulus, trying to connect the two areas of anatomic obstacles. We moved the ablation catheter along this line obtaining local electrogram disappearance or  voltage reduction more than 70%. EnSite NavX mapping system allowed us to move ablation catheter in  the right atrium without using fluoroscopy  during the procedure.

**Figure 2:**
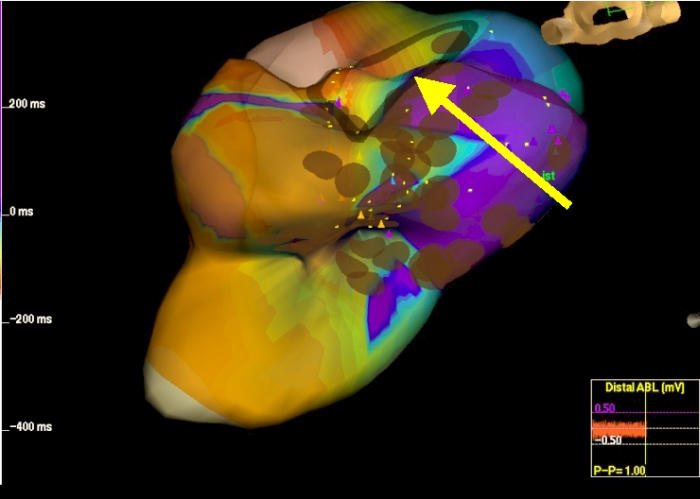
Ablation line remapping showed a gap in the line (yellow arrow).

**Figure 3:**
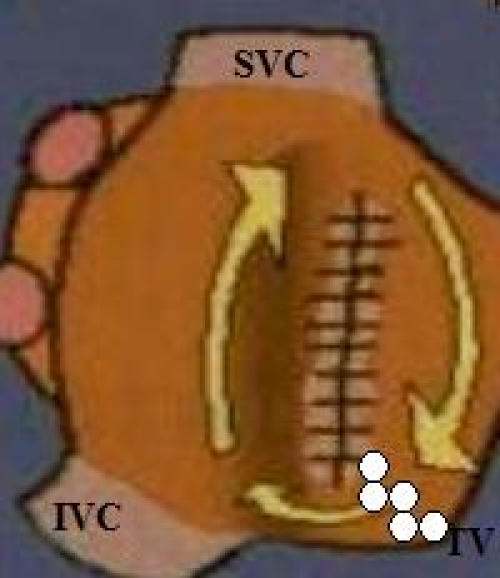
Schematic representation of RA, scar and RF line that interrupted Incisional tachycardia. RF application  in continuous way (70 Watts 55 °C) was attempted drawing back the ablation catheter from the scar area to the tricuspid valve annulus. White points show RF lesion line. (SVC: superior vena cava; IVC: inferior vena cava; TV: tricuspid valve)

**Figure 4:**
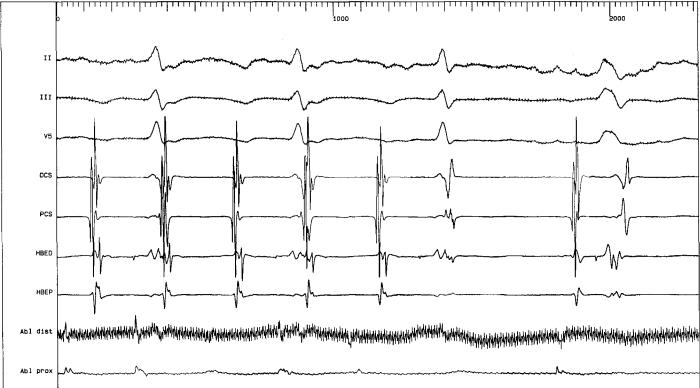
RF delivery along the gap was followed by abrupt interruption of atrial tachycardia with restoration of sinus rhythm.

After four RF applications along the isthmus between the patch on the atrial septum and the tricuspid valve annulus, atrial tachycardia was not yet interrupted though no significant local electrogram was still detectable along the ablation line we had performed. In order to verify ablation line continuity we performed an electroanatomic and activation remapping of the isthmus region which showed a gap on the ablation line (Figure 2) near the tricuspid valve annulus. A unique RF delivery (70 Watts 55 °C) on the gap was followed by abrupt interruption of atrial tachycardia with restoration of sinus rhythm (Figure 3and 4).

## Follow up

The patient was followed up monthly after the procedure. Each month history, 12-lead ECG and 24 hour ECG Holter were analyzed. Ten months after the procedure though the patient had not taken any anti-arrhythmic drugs, there was no recurrence of palpitation or documented atrial tachycardia.

## Discussion and Conclusion

The case shows that EnSite NavX system provides an excellent electroanatomic and activation map of any cardiac chamber and reliable monitoring of the ablation catheter. This system let us quickly made a diagnosis of incisional tachycardia with an anatomic circuit of macroreentry different from that of a common atrial flutter allowing us to identify a slow conduction isthmus in a place that was not the CT isthmus, though the electrocardiography feature of the atrial tachycardia was similar to a common atrial flutter. The Ensite NavX system rapidly identified a gap in the linear lesion we had performed, and allowed us to terminate the atrial tachycardia. NavX significantly reduces fluoroscopy time during ablation procedures and also allows to successfully perform difficult procedures with complex substrates. NavX supports detection of anatomic isthmus variations, particularly deep pouches or recesses [13,14].
